# (Video Assisted) thoracoscopic surgery: Getting started

**DOI:** 10.4103/0972-9941.38912

**Published:** 2007

**Authors:** Tamas F Molnar

**Affiliations:** Department of Surgery, Medical School, University of Pécs, Hungary

**Keywords:** Minimally invasive surgery, rural surgery, surgical techniques, thoracoscopy, video-assisted thoracic surgery

## Abstract

Thoracoscopic surgery without or with video assistance (VATS) is simpler and easier to learn as it seems to be. Potential benefits of the procedure in rural surgical environment are outlined while basic requirements and limitations are listed. Thoracoscopy kit, thoracotomy tray at hand, patient monitoring, proper drainage system, pain control and access to chest physiotherapy are the basic requirements. Having headlight, bronchoscope, Ligasure and mechanical staplers offer clear advantages but they are not indispensable. Exploration and evacuation of pleural space, pleurodesis, surgery for Stage I and II thoracic empyema are evidenced fields of VATS procedures. Some of the cases can be performed under controlled local anesthesia. Acute chest trauma cannot be recommended for VATS treatment. Lung cancer is out of the scope of rural surgery.

## INTRODUCTION

Search for a title which properly explains the aim of a paper and covers the full contents of the article in question is rarely a success story. It is more true, if one intends to outline the very basics of a seemingly highly sophisticated field like thoracoscopic surgery and ends it up in a kind of a cooking book compiled by ingredients. Present day's surgical practice being protocol-bound and evidence-freak, it does not offer a warm invitation for pioneering initiatives. But is thoracoscopic surgery so mystically complicated as it looks like or as it is said to be ? This is an attempt of saying no and yet providing a proper explanation for the attitude presented. The reader is warned in advance against the highly subjective tone and approach which is only partly excusable by the tunnel vision of the author working too long in the pit.

What are the excuses for putting forward the idea of the implementation of certain (video assisted) thoracic surgery (VATS) procedures within a general surgical practice? The scope of the present paper is focused on those circumstances, where the surgeon generally has no unlimited access to advanced medical technologies, due definitely to the the part of the globe where he/she is scrubbing in. The relative unimportance of hardware limitations on the outcomes is very finely reflected and exemplified by the recent echoes of the mesh repair of inguinal hernia.[1]

Thoracic surgery, in general,is not so exclusive at all as it seems to be. General surgeons are sharing definitely a lot more in thoracic surgery than one can imagine according to received wisdom. Behaviour of the pleural space is not so complex and consequently hostile, as one might think impressed by basic physiology and pathology handbooks. Similarily, the lung parenchyma is more forgiving than heralded by its reputation. Aggressive physiotherapy, adequate pain relief and proper chest drainage/suction system are the three pillars of a complication-free post procedural course.

In the United States, a non-negligible number of major thoracic procedures is performed by general surgeons.[2] Certain European countries are also not exceptions. These tendencies are being continually challenged and debated on base of the assumption that the individual patient will benefit more from the higher level of specialization. The difference seems to be obvious and morbidity/mortality data provide evidences. However, budget considerations, questions of access and number of available specialists are limiting factors on the other hand. There are well known and widely accepted territories, such as thoracic sympathectomy, minor peripheral lung procedures (metastectomy) and acute chest trauma where more than one acceptable competence scenario exist. These are fields, where necessities (needs emerging in certain situations) overwrite the hypothetical rule: “chest cavity: enter only for (cardio)thoracic surgeons”. Proper compromises should be made resisting the temptation of over-mystifying thoracic surgical procedures. There are possibilities for specific interventions and there is also room for consideration of performing a certain array of VATS procedures within a general surgical environment.

However, all the while I state this, it has to be stressed that an ideal solution would be a complex cardiothoracic center with a VATS unit in it. Till this Paradise arrives, we have to provide our patients the best care we are able to give. There is no moral reason not to provide, what is possible right now. The concern should be to keep to the proper safety margin in order to protect our patients from surgical excesses and assure the benefits of the available procedures at the same time.

I have a further reason to encourage VATS technique and this is an old hobby horse of mine, the history of our art.[3] Laparoscopy was born as a younger daughter of thoracoscopy nearly a century ago.[4] Thoracoscopy itself was not performed by surgeons (even thoracic surgery as an entity has not been invented yet) but by pneumonologists struggling with tuberculosis. It became a standard and well established procedure well before general anesthesia, widely available and safe. Later on surgeons and gynecologists have infiltrated the battlefield, extending the indications of the procedure. They did it with far less access to teaching material, educational background and professional backup than what we have now.

Thoracoscopy was introduced by chest physicians and reckless surgeons in the era of the White Death, phtysis when arteficial pneumothorax was the only effective treatment available. However, the method did not gain the popularity it deserved not only because no firms pushed hard enough to produce profitable metal tubes with simple optics at their end. In our bright new age, where industry and profit are the motors of progress, VATS became a really lucrative field and the situation has changed completely.

Therefore, I personally would not deter anybody who has basic experience in thoracic surgery from doing thoracoscopic surgery and its special case, where video-assisted thoracic surgery (VATS) is concerned.

Lungs behave like a real ally to surgeon as they have an exceptional potential for healing if supported properly. Clearance of intrabronchial mucus, enforced breathing activity to obtain the maximal possible lung expansion both by aggressive physiotherapy and pain relief are the basic requirements. In many cases of thoracic empyema even the surgery itself can be relegated into the supportive role. Suction system management and physiotherapy are equally important. I found the incentive spirometry indispensable just like in thoracic trauma aftercare.

Saying yes to certain VATS procedures in rural surgery, a structured list of tools and a critical overview of the procedures should be presented.

What are the items of a basic armamentarium without which no self respecting surgeon would start to think about (video assisted) thoracoscopic procedures? The answer includes compulsory elements and suggested items.

### Without what not?

1. Basic thoracotomy tray and thoracoscopy kit
2. Monitors, ie: ECG and pulse oxymetry
3. Access to proper and comitted physiotherapy
4. Properly choosen simple and understandable pleural cavity suction systems
5. Experience and ability to perform a thoracotomy and experience in basic intrathoracic procedures (If one is expected to deal with thoracic trauma: i.e. vessel ligation/suture, stitch and lung tissue handling: i.e. suture and stapling his/her knowledge must be considered sufficient. Alas, these are the required elements of any basic surgical training, therefore no specialist registration should be delivered without them.)
6. Formal or informal contact with a backup institution which is able to cope with the late complications. There is no such a thing as a complications-free surgey, therefore it is advisable to have a secondary or tertiary referral center one can turn to if need arise.

Obtain their trust and secure availability first, rather than test them only when you are in need. Since the times of Hyppocrates amd Shurshuta, the members of the profession have to rely on each other. I only hope that globalization did not erode too much of this discipline. If you establish a “Line of Evacuation” there is a good chance, that you will not need it. Better to say - your patient will not need it. The good old rule: “drains are like insurance: you need them when you do not have them” applies here, too.

### What is useful if is at your disposal?

Apart from the standard operational theatre settings, there are a few additional tools which are really needed.

1. A headlight is advised, as thoracic cavity has much more hidden and dark recesses than the abdomen. Light transmission cable with a good quality light source, preferably the same one can be used for head light which is indispensable if one has to perform thoracic surgery.
2. Choosing the proper hemostatic device is crucial. Coagulators (mono or bipolar), bipolar vessel sealing (Ligasure) or ultrasonic scalpel (Ultracision) are the main options.[5] Working in a multi-profile operational theatre environment makes your decision easier and the utilization of tools more economical. Recent publications support the thoracic application of Ligasure,[6,7] while harmonic scalpel needs further investigations to clarify safety on lung tissue.[8]
3. Availability of staplers is obviously advantegous but not a ‘sine qua non’. I prefer reusable metallic body tools to the throwaway plastic models. This is another field where rural surgery has many to teach ‘mainstream, high tech surgery’. If this latter would have ears to listen…
4. Bronchoscope.

### Learning curve

Once the surgeon gets familiar with the intrapleural situation orientation and more frequent pathologies, he/she can proceed from evacuations to biopsies. To be honest, I am uncertain about the borders of the definite do not venture areas. As any general surgeon is expected to be able to perform uncomplicated lung parenchyma manoeuvres in case of trauma, I think, the same specialist cannot be forbidden to do the same in elective cases. However, I would leave the final decision to the individual surgeon in the specific situation. I am not convinced that minor parenchymal resections should be performed by a non-thoracic specialist on a regular basis, only if no real alternative exist.

What about the strict requirement of airtightness of these procedures? The once so feared deadly mediastinal flutter, the immediate collapse of circulation due to the shifting have proven to be a myth. Patients with modest pulmonary reserve can tolerate these sorts of procedures and temporary communication between the pleural space and the surrounding environment does not lead to serious cardiorespiratory consequences. What is important is a proper and continuous evacuation of air/fluid from the pleural space by drainage postoperatively. There is a temptation of not to leave a chest drain behind following a minor procedure within the chest cavity.[9] Only a very few thoracic surgeons would be brave enough (if this is the proper expression) not to resist this temptation. Agressive physiotherapy (incentive spirometry included) and proper pain relief (opioids) are the main factors of success. An underwater seal (Bülau's vessel or equivalent like Heimlich's valve) is a minimal requirement and active suction postoperatively is generally advisable.

Narcosis of the patient is another important issue. Neither general anesthesia nor selective intubation of the lungs are absolutely necessary. Jacobeus's thoracoscopy patients were awake[4] and thoracic surgeries were performed using peridural anesthesia and opioids in the 1950s without any harm to the patients.[10] Our patients can also benefit from the sophisiticated pain and consciousness control of our days.[11] Pulse oxymetry and additional monitoring systems make old surgical techniques reimplementable in a more sophisticated environment of our present day operational theatres.[12]

## RESULTS OF CONSIDERATIONS

### Definite yes for VATS

Thoracoscopy is proven to be ideal for exploration of the pleural space.[13] Whatever pathology is encountered there, diagnostic and more frequently than not, therapeutic evacuation under direct visual control are possible. Pleurodesis for malignant pleural effusions and pneumothorax is an equally straight forward simple and effective procedure. The good old talc is proven to be the most efficient and cost-effective method so far.[14] Evacuation of hydrothorax of whatever origin, hameothorax and any infectious material (pus) are safely and usually completetely performed in this way. It is worthwile to consider VATS also for the evacuation of Stage I and II thoracic empyema.[15] During changing or repositioning intercostal catheters/tubes, thoracoscopy will provide benefits from direct evacuation and proper positioning of drains. The surgeon, familiar with thoracic drainage can easily perform an exploring thoracoscopy using the same port. If the port hole is properly anesthetised, the procedure can be performed without general anesthesia.[11,12] It is important, to stay the shortest possible time within the thoracic cavity, however never act in a hurry. A sytemic visual scanning of the space in a premeditated way is advisable. What the surgeon needs also, is a long metal suction tube for dissection with a sidehole near the blunt tip at the end to avoid injuries. Curette or Volkmann's spoon are also helpful.

### Definite no's for VATS in a rural setting

1. Acute chest trauma
2. Primary lung cancer

The reasons of my objections are rather based on personal conviction than the highest level evidence based medicine. The primary objective in acute chest trauma is a decision whether a thoracotomy is needed in order to control major bleeding/airway injury or not. A properly performed chest drainage accompanied by close monitoring of the patient/evacuate provides sufficient data for an exploration/observation decision, there is no need to waste time/resources with VATS.

The author of this article firmly believes that VATS would be contraindicated in surgery of primary lung cancer. However considering the costs of 6-8 endo-staplers/operation, at least a double OP time compared to an open procedure and the need for a utility thoracotomy not significantly smaller than a limited axillary thoracotomy the pros for VATS lobectomy/pneumonectomy are far for being obvious.[16,17] On top of these considerations, there is a question of the compulsory hilar/mediastinal lymphnode mapping in cancer surgery, which is not such a straight forward keyhole procedure as it seems to be.

## CONCLUSIONS

There is a huge responsibility on the author of recommending this or that procedure and signalling others as ‘nos’ while reviewing an array of methods in a quite autocratic and dogmatic way as I did it. Based on my teaching experience in thoracic surgery for two decades, I built up an overall view on what can be expected from the average trainee. I did not trespass this hypothetical threshold in this article. However, I cannot avoid to call the attention of the VATSists of the future to the brilliant essay of Ralph J Lewis: The Perils of Pioneering.[18] There is no better summary of the challenges ahead on the field covered in this article than the T Huxley citation by Dr Lewis “It is the customary fate of new truths to begin as heresays”. My humble extension to Mr Huxley's observation is only this: it is worth to try to start walking on this path…
